# X-CAP improves pathogenicity prediction of stopgain variants

**DOI:** 10.1186/s13073-022-01078-y

**Published:** 2022-07-29

**Authors:** Ruchir Rastogi, Peter D. Stenson, David N. Cooper, Gill Bejerano

**Affiliations:** 1 Department of Computer Science, Stanford University, Stanford, USA; 2 Institute of Medical Genetics, Cardiff University, Cardiff, UK; 3 Department of Developmental Biology, Stanford University, Stanford, USA; 4 Department of Pediatrics, Stanford University, Stanford, USA; 5 Department of Biomedical Data Science, Stanford University, Stanford, USA

**Keywords:** Pathogenicity prediction, Stopgain, Nonsense, Machine learning

## Abstract

Stopgain substitutions are the third-largest class of monogenic human disease mutations and often examined first in patient exomes. Existing computational stopgain pathogenicity predictors, however, exhibit poor performance at the high sensitivity required for clinical use. Here, we introduce a new classifier, termed X-CAP, which uses a novel training methodology and unique feature set to improve the AUROC by 18% and decrease the false-positive rate 4-fold on large variant databases. In patient exomes, X-CAP prioritizes causal stopgains better than existing methods do, further illustrating its clinical utility. X-CAP is available at https://github.com/bejerano-lab/X-CAP.

**Supplementary Information:**

The online version contains supplementary material available at (10.1186/s13073-022-01078-y).

## Background

Genome sequencing has revolutionized our ability to diagnose Mendelian diseases [1]. However, individuals contain hundreds of variants of uncertain significance (VUS) within their genomes, and interpreting these variants presents a difficult challenge. Despite the continuous accumulation of known pathogenic and benign variants in databases such as ClinVar [2] and the Human Gene Mutation Database (HGMD) [3], they are far from complete. For example, ClinVar has high-confidence pathogenicity labels for fewer than 100 thousand of all possible 82 million missense variants [4], and the HGMD collection grows by thousands of pathogenic variants every year [3, 5]. This necessitates the development of computational tools that can distinguish pathogenic variants from benign ones. In silico pathogenicity predictors often utilize sequence conservation measures and protein annotations to accomplish this goal. The scores output by these tools are also integrated as valuable features into more holistic models, such as Exomiser [6] and AMELIE [7], that consider patient phenotypes.

**Fig. 1:**
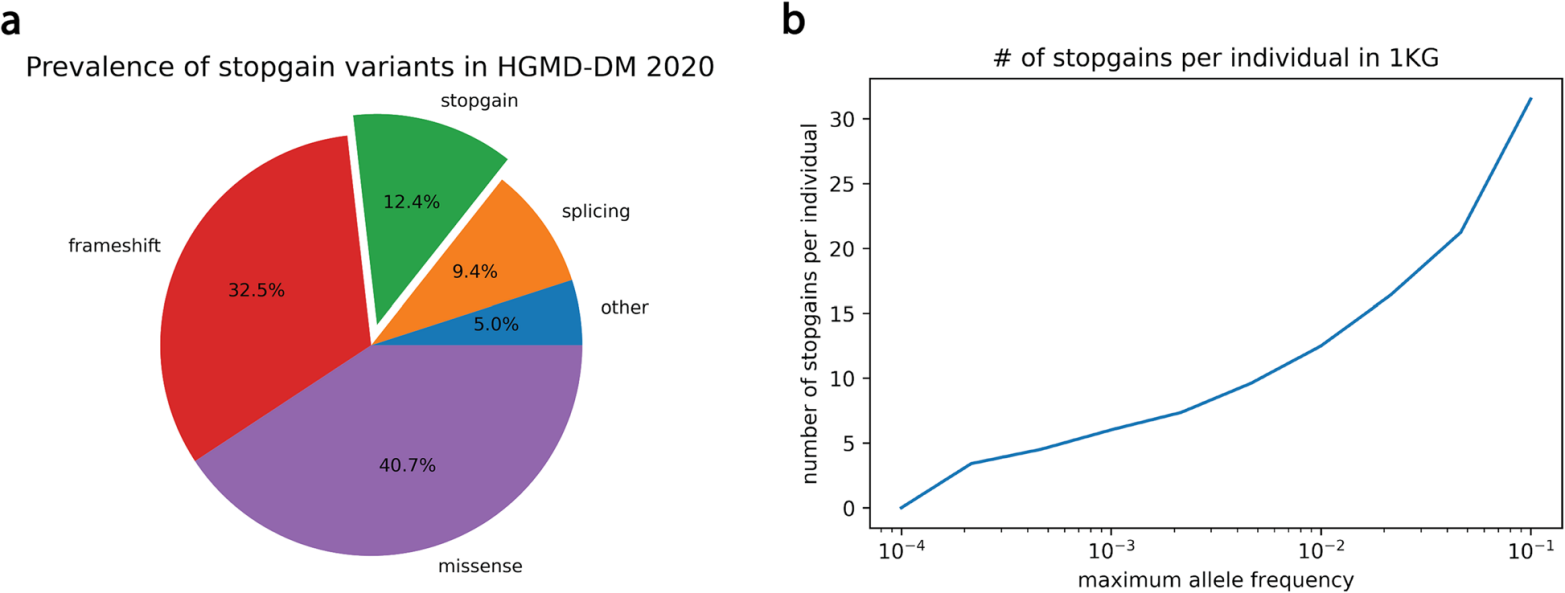
Stopgains are a sizable variant class. **a** The number of variants of each mutation type as a proportion of all DM (disease-causing) variants in HGMD 2020.1. Single base-pair stopgains are the third-largest class, trailing only missense variants and frameshift indels. **b** The prevalence of stopgains from Phase 3 of the 1000 Genomes Project (*N*=2504) as a function of their allele frequencies within the same dataset. The average individual in the dataset harbors 12.5 stopgains with an allele frequency of less than 1%

Historically, pathogenicity predictors, such as M-CAP [8], have focused on missense variants due to the large number of missense VUS within patient exomes [9]. Recently, tools have been developed for noncoding mutations; for example, S-CAP [10] predicts the pathogenicity of splicing mutations. However, other classes of coding mutations, including stopgain mutations, remain understudied. Stopgain substitutions, also called nonsense mutations, prematurely terminate protein translation by converting codons that are normally translated into amino acids into one of three stop codons (TAG, TAA, and TGA). Owing to their large effect on proteins, these mutations have unsurprisingly been implicated in many monogenic disorders, including cystic fibrosis and Duchenne muscular dystrophy, and more complex diseases, such as cancer and neurological disorders [11]. Indeed, single base-pair stopgain substitutions represent the third-largest class of disease-causing variants within HGMD (Fig. 1a) and are often the very first class of variants looked at during patient exome interpretation [12]. However, individuals also contain benign stopgains. Analysis of exomes from the 1000 Genomes Project [13] reveals that the average individual contains more than a dozen rare (allele frequency <1*%*) stopgain substitutions (Fig. 1b). These mutations do not cause monogenic disease for a variety of reasons. Some affect loss-of-function tolerant genes [14]; others preserve protein function due to limited truncation of important domains, stop codon read-through, avoidance of nonsense-mediated decay (NMD), or the use of alternative translation start sites [15]. Since any patient sequenced will have many stopgains and because stopgain pathogenicity is influenced by the complex interaction of many biological factors, computational tools are needed to identify causal mutations.

Whole-genome predictors, such as CADD [16], DANN [17], and Eigen [18], provide pathogenicity scores for all single-nucleotide variants (SNVs), including stopgain mutations, throughout the genome. However, these tools have been engineered for and benchmarked on missense and noncoding variants, not stopgains. Two predictors, MutPred-LoF [19] and ALoFT [20], explicitly focus on stopgain variants. MutPred-LoF and ALoFT input feature representations consisting of evolutionary conservation statistics, protein annotations, and gene essentiality data into an ensemble of two-layer neural networks and random forests, respectively. However, both fail to account for variant zygosity in their prediction pipelines, and their feature sets do not capture several intricacies of stopgain-specific biology. Moreover, neither is calibrated to perform well in the high-sensitivity region [8]—the performance regime in which a model attains a sensitivity of 95% or more, which is required in a clinical setting (see below).

In this paper, we introduce X-CAP, a conceptual sequel to M-CAP (missense) [8] and S-CAP (splicing) [10], that addresses the aforementioned shortcomings of existing stopgain pathogenicity predictors. We evaluate X-CAP at the high sensitivity required in clinical settings and show that X-CAP considerably outperforms existing methods. The X-CAP source code and predictions for all human stopgain substitutions can be found at https://github.com/bejerano-lab/X-CAP [21].

## Methods

We developed a machine learning framework to predict the pathogenicity of stopgain substitutions. This involved (a) curating two labeled datasets of benign and pathogenic stopgain variants, (b) designing a set of informative features that discriminate between the two classes, and (c) learning a model that performs well at high sensitivity. We show that X-CAP boasts superior performance when evaluated on the aforementioned datasets, as well as on patient exomes.

### Dataset curation

To assemble the first dataset (named $\mathcal {D}_{\text {original}}$), we incorporated pathogenic variants from the 2019.1 Professional version of the Human Gene Mutation Database (HGMD), which curates inherited pathogenic variants from the peer-reviewed literature [3], and putatively benign variants from the 2.1.1 exomes version of the Genome Aggregation Database (gnomAD), which curates sequencing data from individuals not known to be affected by a Mendelian disease [14].

We isolated single base-pair stopgain substitutions using ANNOVAR [22] and included, in both the pathogenic and benign sets, only those variants with an allele frequency less than 1%. Rare variants were isolated because most pathogenic monogenic mutations affect less than 1% of the population [23], and, therefore, the American College of Medical Genetics and Genomics recommends that more common variants be deemed non-causative [24]. This recommendation is well-supported by our data, as only 4 out of 25,098 (0.016%) pathogenic stopgains in HGMD have an allele frequency greater than this threshold. Moreover, removing common benign variants is beneficial because models trained on datasets that retain them tend to poorly distinguish rare benign variants from pathogenic variants [25]. After this filtration step, 25,094 pathogenic and 160,247 benign stopgains remained. We randomly split those variants into training and test sets, ensuring that variants used by MutPred-LoF [19] or ALoFT [20] (either within their training or test sets, as their exact splits could not be obtained) were routed to our training set. (CADD [16], DANN [17], and Eigen [18] do not train on known pathogenic or known rare benign variants, so there is no overlap between their training datasets and ours.) Additional file 1: Fig. S1a summarizes our pipeline.

When generating the dataset, we considered a variant *v* to be a 5-tuple of (chrom, pos, ref, alt, zygosity). In particular, variants at the same locus could have conflicting pathogenicity labels if their zygosities differed. We consider this to be a strength of our design, as it allowed the model to learn a decision boundary between variants that are pathogenic as homozygotes but benign as heterozygotes.

To evaluate the robustness of our model, we also assembled $\mathcal {D}_{\text {validation}}$, which contains novel benign stopgains from gnomAD genomes 3.0 and pathogenic variants from HGMD Professional 2020.1 and ClinVar [2]. The same pipeline described above was used to filter rare stopgains, and those variants contained in $\mathcal {D}_{\text {original}}$ or seen by other tools were discarded (Additional file 1: Fig. S1b). After filtration, 10,295 pathogenic variants and 53,622 benign variants remained.

Two additional datasets containing patient variants were also constructed. First, we collected rare, putatively benign stopgains from patient exomes in a control cohort (*N*=480) of an Inflammatory Bowel Disease study (dbGaP Study Accession: phs001076.v1.p1, consent group: GRU) [26]. Second, we sourced causal pathogenic stopgains from patients in the Deciphering Developmental Disorders project [27] who harbored one stopgain and no other rare mutations in the causal gene. For both patient datasets, variants contained in $\mathcal {D}_{\text {original}}$ or $\mathcal {D}_{\text {validation}}$ and variants seen by other classifiers were discarded.

### X-CAP features

**Fig. 2:**
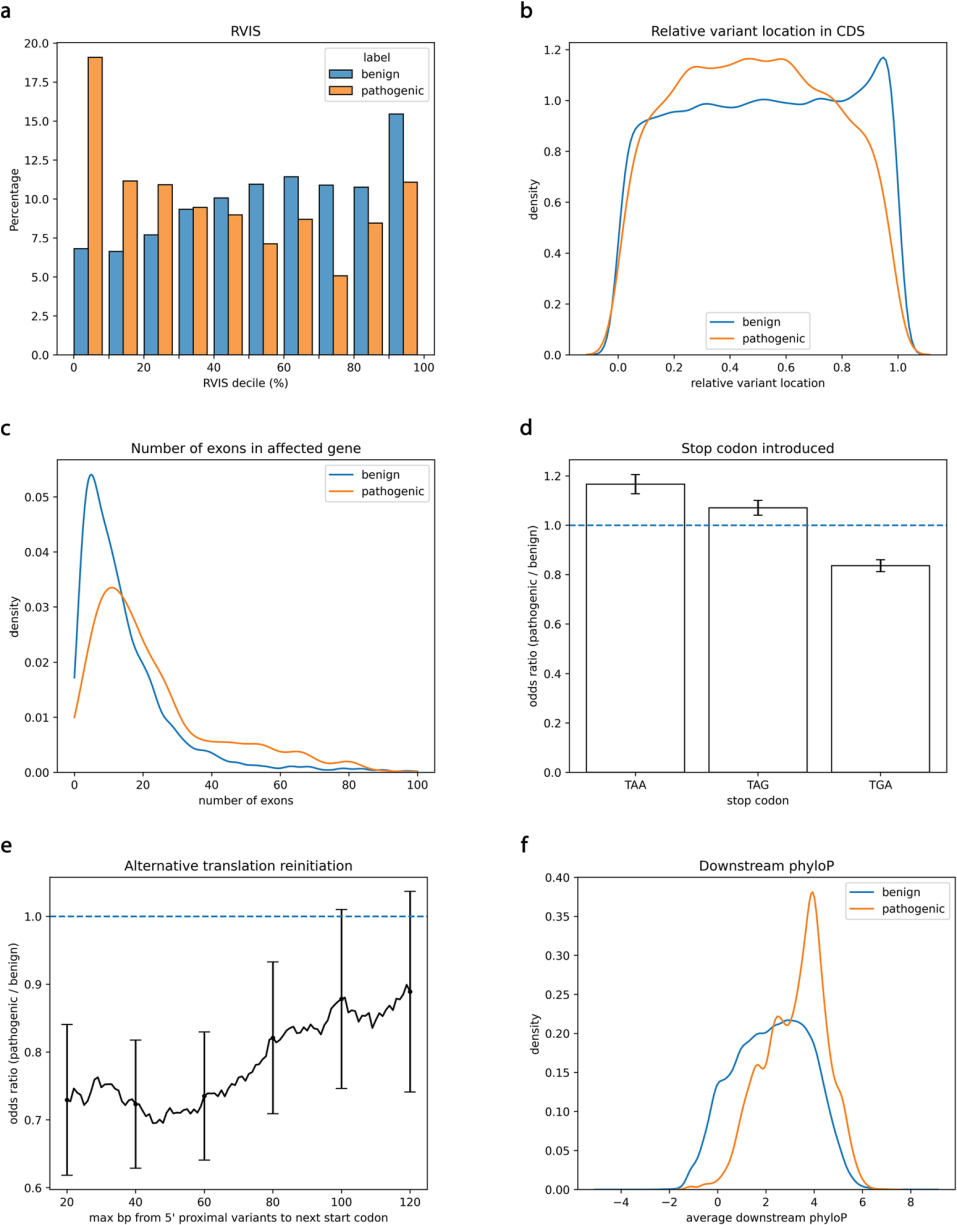
X-CAP features show predictive power. Comparison of feature values for benign and pathogenic stopgains in the training set of $\mathcal {D}_{\text {original}}$. **a** The Residual Variation Intoleration Score (RVIS) decile of genes, weighted by the number of variants they contain. Genes without RVIS values were excluded. Pathogenic variants are more prevalent in low RVIS genes, namely those generally intolerant to variation. **b** Kernel Density Estimation (KDE) plot of the relative variant location, defined as the distance in the coding domain sequence (CDS) from the translation start site divided by the total CDS length. On average, benign stopgains are located later in transcripts than pathogenic stopgains. **c** KDE plot of the number of exons in the mutated gene. The maximum number of exons is clipped to 100 for clarity. Genes containing benign stopgains tend to have fewer exons than genes containing pathogenic stopgains. **d** Odds ratios (pathogenic/benign) comparing variants that introduce a given stop codon to those that do not. The TGA stop codon, molecularly shown to be the most amenable to read-through of the three [36], is depleted in pathogenic variants. **e** Odds ratios comparing 5’ proximal stopgains (those within the first 100 bp of the sequence) that have a potential alternative downstream start codon a given distance away against those that do not. Pathogenic variants tend to be located further from the next downstream start codon than benign variants. **f** KDE plot of the mean phyloP of the downstream region, the portion of the CDS truncated by the stopgain. Regions downstream of pathogenic variants are more conserved than regions downstream of benign variants. In **b**, **c**, and **f**, Scott’s Rule [52] was used to calculate the bandwidth of the Gaussian kernel. In **d** and **e**, error bars denote 95% confidence intervals for the odds ratio

Predicting a stopgain’s pathogenicity reduces to two questions. First, does the stopgain significantly alter the resulting protein? Second, if it does, can one or two copies of the abnormal protein be tolerated? Existing classifiers tend to focus on one of these two questions, but not both: MutPred-LoF focuses on the former, whereas ALoFT focuses on the latter. To address both questions simultaneously, we included the variant’s zygosity, measures of gene and exon essentiality, and stopgain-specific features. For any feature that could vary across transcripts, we took an average over the transcripts that the variant overlaps. Table 1 summarizes all features used by X-CAP, Fig. 2 shows the separation power of select features, and more implementation details are included within Additional file 1: Supplementary Methods.

**Table 1:** X-CAP features. *violetItalicized features* are novel and have not been used in previous stopgain pathogenicity predictors. Specifically, no features related to zygosity, stop codon read-through, or alternative translation reinitiation are present in earlier classifiers

Feature type	Feature name	Description
Zygosity	*violetzygosity*	Binary variable distinguishing homozygous (and hemizygous) variants from heterozygous variants, inputed when known or predicted as a function of benign stopgain alleles at the same position in training set when unknown
Gene/exon essentiality	*violetoe*	Number of benign stopgains in training set along gene divided by gnomAD’s expected number of loss-of-function variants
	RVIS	Measure of gene intolerance to functional variation
	OMIM gene map	Two non-exclusive, binary features indicating whether a recessive or dominant disease listed in the OMIM Gene Map is caused by mutations in this gene
	*violetmonoclass pathogenic*	Transcript or exon contains no benign variants and at least one pathogenic variant within training set
	*violetcan be spliced out*	Variant is skipped in at least one isoform of the gene
Variant location	distance from CDS start/end	Number of coding nucleotides from CDS start and end
	relative CDS location	Distance from CDS start divided by CDS length
	*violetdistance from exon start/end*	Number of coding nucleotides from exon start and end
	*violetrelative exon location*	Distance from exon start divided by exon length
	*violetexon length*	Number of nucleotides in overlapped exon
	*violetexon number*	Index of the exon that the variant overlaps
	*violet# transcript exons*	Number of exons in overlapped transcript
	chromosome	Ternary variable indicating if the variable is located on an autosomal, X, or Y chromosome
NMD	distance from last exon-exon junction	Number of coding nucleotides upstream from last exon-exon junction (negative if downstream of junction)
	*violet% transcripts with NMD*	Percentage of overlapped transcripts in which the variant is >50 bp upstream of the last exon-exon junction
Stop codon read-through	*violetstop codon*	One-hot encoding of the new stop codon introduced by the stopgain
Alternative translation reinitiation	*violetdistance to next start codon*	Number of base pairs between the variant and the next potential downstream start codon within the mRNA
Cross-species conservation	phyloP	Base-pair conservation across vertebrates of upstream, downstream, and overlapped exon regions
	phastCons	Regional conservation across vertebrates of upstream, downstream, and overlapped exon regions

#### Zygosity

In patients, sequencing reveals the zygosity of each variant. This information is crucial in determining pathogenicity, as one normal copy of the gene could be sufficient to prevent monogenic disease. Indeed, in our dataset, 8736 pathogenic stopgains from HGMD had benign heterozygous counterparts in gnomAD, revealing that zygosity strongly influences pathogenicity. While gnomAD includes the zygosity of its variants, HGMD and ClinVar do not. Thus, for pathogenic variants with unknown zygosity, we employed the following heuristic. If the pathogenic variant was present within gnomAD in a heterozygous state, we predicted it to be homozygous; otherwise, we predicted it was heterozygous. Note that this prediction is internal to our model, which ultimately outputs pathogenicity scores for variants either with or without known zygosity (see Discussion).

#### Gene/exon essentiality

We included various features that serve to capture the essentiality [28] of the affected gene and exon. First, we derived a stopgain-specific version of gnomAD’s *oe* (observed/expected) ratio [14] in order to quantify a gene’s intolerance to stopgain mutations. We also supplied RVIS [29] values (Fig. 2a) and noted if a given gene was implicated in a recessive or dominant Mendelian disease (or both), as cataloged in the Online Mendelian Inheritance in Man (OMIM) Gene Map [30]. Additionally, we classified transcripts and exons as monoclass pathogenic if at least one pathogenic variant, but no benign variants, was present along the transcript or exon within the training set. We did not classify transcripts or exons by the lack of pathogenic variants because hundreds of novel monogenic disease genes are discovered every year [5, 31]. Lastly, to allow for alternative splicing, we checked if the stopgain was skipped by any isoform of the gene [32].

#### Stopgain-specific features

These features can be divided into five categories: variant location, nonsense-mediated decay, stop codon read-through, alternative translation reinitiation, and cross-species sequence conservation.

First, we included the location of a stopgain within its transcript in order to estimate the extent of damage caused by premature truncation. Pathogenic variants truncated slightly more of the sequence than benign variants (51% v. 48% on average, *P*<10^−58^ by one-sided Welch’s *t*-test). Notably, pathogenic variants were depleted near the end of the sequence (Fig. 2b). Variants near the end may not significantly disrupt protein function or may avoid the effects of nonsense-mediated decay (NMD; see below). We also created features for the number of exons in the mutated transcript and the index of the exon affected by the stopgain. Interestingly, benign stopgains were located on transcripts with fewer exons than those pathogenic stopgains were on (15.5 v. 25.1 on average, *P*<10^−306^ by one-sided Welch’s t-test; Fig. 2c).

NMD is a pathway by which mRNAs containing premature stop codons are degraded before translation [33]. NMD is predicted to be triggered when the premature stop codon is more than 50 base pairs upstream of the last exon-exon junction [34]. We included the distance to the last exon-exon junction and the percentage of transcripts in which NMD is predicted to occur as features.

Stop codon read-through occurs when the ribosome continues translating past the stop codon, and drugs that promote read-through are commonly used to treat diseases caused by stopgains [35]. Experimental evidence in mammalian cells indicates that the three stop codons have different read-through rates with TGA>TAG>TAA with respect to the likelihood of read-through [36]. In concordance with these molecular results, we found that the TGA stop codon was depleted in pathogenic variants, whereas the TAG and TAA stop codons were enriched (largest *Q*<10^−4^ after a Bonferroni correction to the Pearson’s chi-squared test; Fig. 2d).

Alternative translation reinitiation allows for the circumvention of 5’ proximal stopgains [37] if there are potential start codons downstream. The efficacy of this circumvention depends not only on the distance between the translation start site and the variant but also on the distance between the variant and the next start codon [38], so both distances were included as features. The benign set was found to be enriched for stopgains that were close to downstream start codons, and, as expected, the strength of that enrichment was inversely correlated with the distance to the downstream start codon (Fig. 2e).

Lastly, we included phyloP [39] and phastCons [40] scores from multiz100way alignments of vertebrates [41] to measure the evolutionary conservation of the truncated region. On average, the regions downstream of pathogenic variants were more conserved than the regions downstream of benign variants (Fig. 2f).

### X-CAP’s learning algorithm

X-CAP uses a gradient boosting tree (GBT) classifier to discriminate pathogenic stopgains from benign ones. In a GBT model, a collection of decision trees is iteratively assembled. Each decision tree predicts the residual unaccounted for by the previous trees, and the final classifier is a weighted linear combination of each of the previously derived decision trees [42]. Fivefold cross-validation was used to select features and tune hyperparameters (see Additional file 1: Supplementary Methods). To understand the importance of X-CAP’s features, we computed Shapley values using the shap package [43].

### Model comparison

We compared our method to ALoFT [20], MutPred-LoF [19], CADD [16], DANN [17], and Eigen [18] on the aforementioned datasets. ALoFT was run after lifting over variants to the hg19 assembly using the LiftoverVcf command from the Picard tool suite [44]. MutPred-LoF was run using the output of ANNOVAR’s coding_change.pl script as input. Because of the long running time of the model (MutPred-LoF is 84 times slower than X-CAP on 1000 variants; Additional file 1: Table S1), we randomly subsampled 1000 variants when evaluating it on $\mathcal {D}_{\text {original}}$ and $\mathcal {D}_{\text {validation}}$. CADD, DANN, and Eigen scores were taken from dbNSFP v4.1a [45]. Variants without provided scores in dbNSFP were assigned a default score of 0, which is the label of the benign class.

We assessed each model’s area under the receiver operating characteristic (AUROC) curve and area under the precision recall curve (AUPRC) on $\mathcal {D}_{\text {original}}$ and $\mathcal {D}_{\text {validation}}$. As described further within the “Results” section, we also highlight each model’s AUROC in the clinically relevant high-sensitivity region (true positive rate ≥95*%*). AUROC and AUPRC metrics were computed using the scikit-learn package [46].

## Results

### X-CAP outperforms competitors at clinically relevant thresholds

**Fig. 3:**
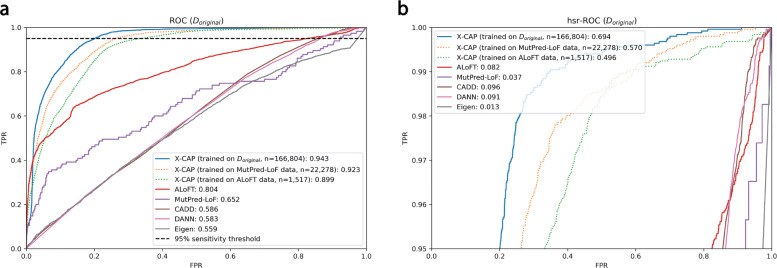
X-CAP outperforms competitors. **a** For each model, we plot the ROC curve and associated AUROC metric on the test set of $\mathcal {D}_{\text {original}}$. X-CAP has the highest AUROC, improving upon the previous state-of-the-art by 0.14 absolute points. The orange and green dotted lines display X-CAP’s performance when trained only on variants present in the databases used by MutPred-LoF and ALoFT, respectively. To ensure a fair comparison, we randomly subsampled these datasets to the size used in the original papers (*n* indicates the size of the training set). **b** We enlarge the portion of the plot above the dashed line in panel **a** to show performance in the clinically relevant, high-sensitivity region (TPR ≥0.95). We also display the hsr-AUROC, which is the normalized area under the curve in the high-sensitivity region. We optimized X-CAP to excel in this region, rather than over the full ROC. At 95% sensitivity, X-CAP correctly classifies 80.0% of benign stopgain variants, over four times more than any other classifier

We compared X-CAP to existing methods on the test set of $\mathcal {D}_{\text {original}}$ (Additional file 1: Fig. S1a). Performance was first measured by examining the area under the receiver operating characteristic (AUROC) curve. X-CAP appreciably improved the AUROC from 0.80 to 0.94 (Fig. 3a). Because of class imbalance in our test set, we also measured the area under the precision recall curve (AUPRC). X-CAP performs best on that metric as well, increasing the AUPRC from 0.57 to 0.68 (Additional file 1: Fig. S2). On both metrics, ALoFT was the second best classifier, and the whole-genome predictors performed worse than any of the stopgain-specific classifiers.

AUROC and AUPRC measure a model’s aggregated performance across all possible decision rules. In this setting, a decision rule maps a variant’s pathogenicity score to a label ∈{benign, pathogenic}. However, a model should primarily be evaluated using the decision rule that will be employed in practice. As argued in M-CAP [8] and S-CAP [10], a clinically useful decision rule must limit false negatives because there is little utility in reducing the size of the candidate list of VUS if the pathogenic variant is incorrectly discarded. Accordingly, we propose a decision rule that achieves 95% sensitivity (recall, true positive rate). The requisite threshold for X-CAP to achieve this is 0.0601. This differs from the suggestions given by MutPred-LoF and ALoFT. MutPred-LoF recommends a decision rule with a 5% false-positive rate. ALoFT’s decision rule assigns the label of the class (one of benign, pathogenic dominant, or pathogenic recessive) with the highest probability. Neither provides any guarantees as to the true positive rate.

Accordingly, we examined the performance of all classifiers in the high-sensitivity region (hsr), the portion of each classifier’s ROC curve in which the classifier’s true-positive rate is greater than 95% (above the dashed line in Fig. 3a). We computed the area under the curve within that region (hsr-AUROC) and found that X-CAP vastly improved performance (Fig. 3b). X-CAP increased the hsr-AUROC by 0.61 absolute points, a nearly 9-fold improvement, and correctly classified 80.0% of benign variants at 95% sensitivity. ALoFT—the next best model—only correctly classified 17.6% of benign variants at the same sensitivity.

To explicitly quantify the impact of X-CAP’s featurization and training methodology, we retrained X-CAP using only variants in $\mathcal {D}_{\text {original}}$ also present in the databases utilized by MutPred-LoF and ALoFT. We ensured that our training datasets were of the same size as those in the original papers to ensure a fair comparison. Even when trained on these older and smaller datasets, X-CAP significantly outperformed both methods (see Fig. 3 legends). Nonetheless, training on additional variant data does further improve X-CAP performance.

### X-CAP generalizes to other variant databases

To ensure that X-CAP is robust to distribution shifts and generalizes well, we evaluated our classifier on a second dataset, aptly termed $\mathcal {D}_{\text {validation}}$. This dataset contains newly discovered benign stopgains in gnomAD genomes 3.0 and pathogenic stopgains in HGMD 2020.1. It also contains pathogenic stopgains from ClinVar, which has a different curation strategy than HGMD.

Despite this distribution shift, the performance of all tools and, in particular, the marked improvement that X-CAP brings is nearly identical on $\mathcal {D}_{\text {validation}}$ (compare Fig. 3 to Additional file 1: Fig. S3 and Additional file 1: Fig. S2 to Additional file 1: Fig. S4) in terms of the overall AUROC, AUPRC, and hsr-AUROC, with almost a 6-fold improvement in the last. The stability of X-CAP’s performance indicates that the model generalizes well.

### X-CAP outperforms competitors on patient data

**Fig. 4:**
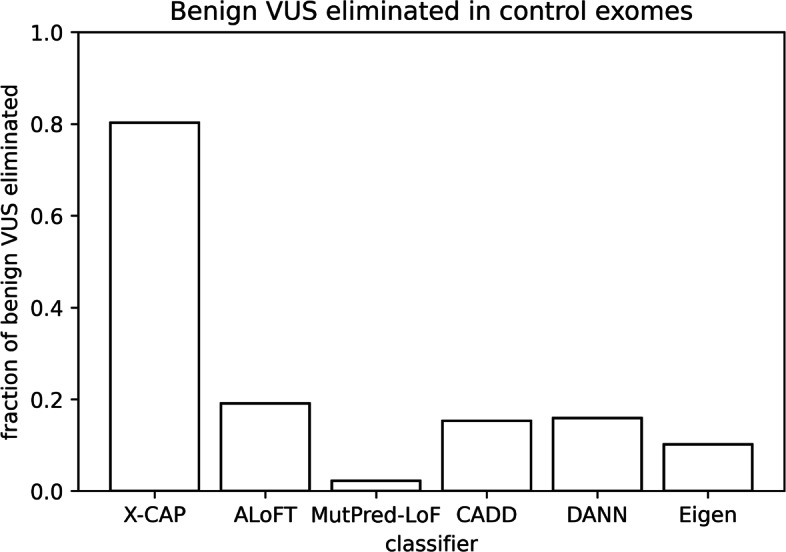
X-CAP eliminates the most benign stopgain VUS in control exomes. We plot the fraction of rare benign stopgain variants that were assigned scores below the 95%-sensitivity threshold for each classifier. These variants were taken from exomes from a control population (*N*=480) in an Inflammatory Bowel Disease (IBD) study. The performance of all classifiers on exomes nicely matches their performance on aggregated variant sets in Fig. 3b and Additional file 1: Fig. S3b. X-CAP increases the percentage of benign VUS eliminated by 4.4-fold

Although tools such as X-CAP are trained on large datasets of pathogenic and benign variants, in practice they are used to reduce the number of VUS in individual patients by identifying likely benign variants. Since patients with monogenic disease conceptually differ from other individuals by only 1 to 2 pathogenic variants, we used a large control population of individuals as a proxy for undiagnosed patients without a causal stopgain mutation. Specifically, we sourced 480 exomes from a control cohort in an Inflammatory Bowel Disease (IBD) exome sequencing study [26] and removed both common variants and those variants previously seen by any classifier. After calibrating each model to achieve 95% sensitivity, we found that X-CAP eliminated 80.2% of benign variants, which is 4.2-fold more than the next best classifier (Fig. 4). These numbers are also very consistent with the true-negative rates observed in Fig. 3b and Additional file 1: Fig. S3b.

Ultimately, we would like these tools to provide higher scores to disease-causing stopgains in patient exomes. To test this, we collected causal stopgains from 10 patients in the Deciphering Developmental Disorders (DDD) project [27]. Table 2 displays the score that each classifier assigned to the causal variants. In six out of ten cases, X-CAP assigned the highest percentile score, whereas no other classifier did so more than once. Moreover, this test vividly demonstrates the importance of calibration for clinical use. If the decision rules originally recommended by each tool were to be used, MutPred-LoF would have mischaracterized the disease-causing variant five times, and ALoFT three times. Thanks to careful calibration, X-CAP made only one such mistake.

**Table 2:** X-CAP prioritizes causal stopgains in patient exomes. Each row in the table describes a single patient, the causative gene and variant, the genotype of the variant, and the percentile-normalized score provided by each classifier. For each method, raw scores were percentile-normalized in comparison to the scores output by the classifier on the test set of $\mathcal {D}_{\text {original}}$. All ten patients contain one rare stopgain and no other rare mutations in the causal gene. **violetBolded entries** have the highest percentile for a given variant. *redItalicized entries* would have been misclassified on the basis of the original authors’ recommendations (CADD, DANN, and Eigen do not provide a decision rule). X-CAP assigns the highest percentile six out of the ten times and mischaracterizes only one variant. No other tool assigns the highest percentile-normalized score more than once, and MutPred-LoF and ALoFT mischaracterize variants five and three times, respectively

Patient ID	Gene	HGVS	GT	X-CAP	MutPred-LoF	ALoFT	CADD	DANN	Eigen
DDDP108441	*FOXP1*	c.C1366T:p.Q456X	0/1	89.4	87.7	89.4	89.2	81.3	**violet95.1**
DDDP108556	*MED12*	c.C5916A:p.Y1972X	0/1	90.8	red*26.6*	**violet93.3**	23.7	52.0	8.9
DDDP108105	*SATB2*	c.C1375T:p.R459X	0/1	**violet98.8**	77.3	96.4	57.2	64.1	70.0
DDDP109873	*EP300*	c.C5581T:p.Q1861X	0/1	90.8	**violet98.9**	98.8	57.2	44.1	46.5
DDDP111266	*CASK*	c.C613T:p.R205X	1/1	**violet99.1**	*red17.9*	97.5	46.0	81.3	6.8
DDDP107416	*AUTS2*	c.C976T:p.Q326X	0/1	*red54.0*	78.6	*red67.4*	17.9	**violet81.3**	45.3
DDDP108492	*DYRK1A*	c.C691T:p.R231X	0/1	**violet98.7**	*red19.5*	90.7	57.2	81.3	70.7
DDDP100091	*KDM6A*	c.C3047A:p.S1016X	0/1	**violet93.6**	*red30.8*	93.1	66.0	64.1	11.2
DDDP110976	*POGZ*	c.T2579A:p.L860X	0/1	**violet93.3**	*red22.9*	*red79.0*	12.3	14.4	25.2
DDDP110748	*ANKRD11*	c.C1801T:p.R601X	0/1	**violet92.6**	83.2	*red69.5*	10.0	21.0	15.2

## Discussion

Single base-pair stopgain substitutions comprise the third-largest class of disease-causing mutations (Fig. 1a); however, only a fraction of stopgains can be assumed to be pathogenic as the average individual contains upwards of twelve rare stopgains (Fig. 1b). X-CAP helps advance the state of the art in stopgain pathogenicity prediction. X-CAP is a calibrated machine learning model that, at 95% sensitivity, correctly classifies more than 80% of rare benign variants (Fig. 3b and Additional file 1: Fig. S3b), four times more than the previous best model. Concretely, for the average patient with twelve rare benign stopgains, X-CAP can greatly downgrade interest in nine to ten while still retaining any pathogenic mutation with very high probability. Moreover, X-CAP provides higher scores to disease-causing stopgains (Table 2) than other models do, so clinicians can use our model to more confidently identify causal variants. X-CAP performs consistently well even on the latest discoveries (such as the new pathogenic stopgains added in HGMD 2020.1 and included in $\mathcal {D}_{\text {validation}}$), suggesting it could have assisted in accelerating their discovery.

The GBT model powering X-CAP, along with our careful featurization, makes X-CAP extremely robust. For example, X-CAP maintains strong performance on variants that are present in genes which were unobserved during training (Additional file 1: Fig. S5). Our model’s performance is also consistent irrespective of the number of transcripts that a variant overlaps (Additional file 1: Fig. S6). And if we rectify the class imbalance in X-CAP’s training set (144,420 benign stopgains vs. 22,584 pathogenic stopgains) by randomly subsampling the benign class, performance only decreases slightly (Additional file 1: Fig. S7).

Feature analysis (Additional file 1: Fig. S8) reveals how our different features come together to contribute to X-CAP’s performance. In particular, inspired by S-CAP’s distinct dominant and recessive classifiers for core splicing variants [10], we set out to explicitly model the zygosity of stopgain variants. While 1000 Genomes, ExAC, gnomAD, and certainly real patient sequencing data come with zygosity, both HGMD and ClinVar choose not to provide the zygosity of pathogenic variants. To address this issue, we predict the zygosity of pathogenic variants from our training data, thereby allowing X-CAP to predict pathogenicity of variants whether their zygosity is given (always preferred) or not. Ablating this (internal) feature modestly reduces X-CAP performance across the ROC curve (Additional file 1: Fig. S9). In the future, our heuristic could be bolstered by extending natural language processing tools, such as AVADA [47], to extract true zygosity tags of curated pathogenic variants directly from the primary literature. Other methodological improvements over ALoFT and MutPred-LOF that we introduce include (1) limiting training to rare variants, (2) incorporating benign heterozygous stopgains within the training set, and (3) performing hyperparameter tuning and feature selection based on performance at high sensitivity as opposed to the overall AUROC.

Aside from zygosity, X-CAP also integrates novel features related to nonsense-mediated decay, stop codon read-through, and alternative translation reinitiation. Many of these features have high importance scores, indicating that they are integral to the model’s decision-making process (Additional file 1: Fig. S8). Our current development of these stopgain-specific features has been guided by general trends observed in molecular experiments. However, as individual-level RNA-Seq [48] and Cap Analysis of Gene Expression (CAGE) [49] datasets are assembled, deep learning tools, similar to LaBranchoR [50] and SpliceAI [51], can be trained to predict these phenomena directly from sequences. These predictions could then easily be added as features into our model to potentially improve performance. It is tempting to consider extending our stopgain substitution predictor to cover frameshifting mutations, as they too often result in premature stop codons. However, because frameshifting mutations result in hard to predict, variable-length amino acid sequence disruptions, we feel a rather different feature library will need to be constructed to optimize performance.

The aforementioned improvements make X-CAP extremely powerful and well adapted to clinical practice, where stopgains are often the first variants to be inspected. X-CAP is also extremely valuable as a high-quality feature in more comprehensive systems, such as AMELIE [7], that integrate pathogenicity prediction tools and supporting literature evidence for patient variants to provide cheap, accessible, democratized, automated patient diagnoses.

## Conclusions

Stopgain variants are an important and understudied class of mutations. In the clinic, there is need for computational tools to identify pathogenic stopgains. Here, we presented X-CAP, a calibrated machine learning model that incorporates variant zygosity, measures of gene and exon essentiality, and novel stopgain-specific features to predict pathogenicity. X-CAP significantly outperforms previous models, particularly in the clinically relevant high-sensitivity region. Additional analysis of our model’s performance on patient exomes suggests that it can provide a transformative clinical impact. Predictions for all stopgains in the human proteome and source code to run X-CAP on specific variants are available at https://github.com/bejerano-lab/X-CAP[21].

## Supplementary Information

**Additional file 1** Supplementary methods, supplementary figures (Fig. S1-S9), and supplementary table (Table S1).

## Data Availability

The X-CAP source code, training and testing variants, and predictions for all human stopgains are available at https://github.com/bejerano-lab/X-CAP. The public version of HGMD [3] is available to users from academic institutions and non-profit organizations at http://www.hgmd.cf.ac.uk/ac/index.php. gnomAD [14] is publicly available at https://gnomad.broadinstitute.org/downloads. ClinVar [2] variants can be downloaded from https://ftp.ncbi.nlm.nih.gov/pub/clinvar/vcf_GRCh38/. Data from the Deciphering Developmental Disorders project [27] can be requested from the European Genome-phenome Archive (Study ID: EGAS00001000775) and is located at https://www.ebi.ac.uk/ega/studies/EGAS00001000775. Access to the Inflammmatory Bowel Disease Exome Sequencing Study data [26] (https://www.ncbi.nlm.nih.gov/projects/gap/cgi-bin/study.cgi?study_id=phs001076.v1.p1) requires authorized access from dbGaP. URLs for feature files used by X-CAP: • gnomAD *oe* [14]: per transcript values at https://storage.googleapis.com/gcp-public-data--gnomad/release/2.1.1/constraint/gnomad.v2.1.1.lof_metrics.by_transcript.txt.bgzand per gene values at https://storage.googleapis.com/gcp-public-data--gnomad/release/2.1.1/constraint/gnomad.v2.1.1.lof_metrics.by_gene.txt.bgz • RVIS [29]: https://genic-intolerance.org/data/RVIS_Unpublished_ExACv2_March2017.txt • OMIM gene map data [30]: https://www.omim.org/search/advanced/geneMap URLs for other tools: • MutPred-LoF [19]: http://mutpred2.mutdb.org/mutpredlof/ • ALoFT [20]: http://aloft.gersteinlab.org/ • ANNOVAR [22]: https://annovar.openbioinformatics.org/en/latest/ • Picard [44]: https://github.com/broadinstitute/picard • shap [43]: https://github.com/slundberg/shap

## Abbreviations

AUPRC: Area under the precision recall curve
AUROC: Area under the receiver operating characteristic
CDS: Coding DNA sequence
dbGaP: Database of Genotypes and Phenotypes
HGMD: Human Gene Mutatation Database
hsr-AUROC: High-sensitivity region area under the receiver operating curve
NMD: Nonsense-mediated decay
OMIM: Online Mendelian Inheritance in Man
RVIS: Residual Variation Intolerance Score
VUS: Variant of uncertain significance
