# Supplementary material for: X-CAP improves pathogenicity prediction of stopgain variants
Source: Genome Med. 2022 Jul 29;14:81. doi: 10.1186/s13073-022-01078-y (PMC9338606; doi:10.1186/s13073-022-01078-y)
Supplement: Supplementary file 1 — Additional file 1 Supplementary methods, supplementary figures (Fig. S1-S9), and supplementary table (Table S1). [file 13073_2022_1078_MOESM1_ESM.pdf]

Supplement for "X-CAP improves pathogenicity prediction of stopgain substitutions"

Ruchir Rastogi[1], Peter D. Stenson[2], David N. Cooper[2], and Gill Bejerano[1,3,4,5,*]

[1]Department of Computer Science, Stanford University, Stanford, CA, USA
[2]Institute of Medical Genetics, Cardiff University, Cardiff, UK
[3]Department of Developmental Biology, Stanford University, Stanford, CA, USA
[4]Department of Pediatrics, Stanford University, Stanford, CA, USA
[5]Department of Biomedical Data Science, Stanford University, Stanford, CA, USA
[*]Corresponding Author: bejerano@stanford.edu

1 Supplementary Methods

Prevalence of stopgain variants

The number of pathogenic mutations of each class in HGMD Professional 2020.1 [1] was counted by examining the `mutype` tag for all DM (disease-causing mutation) variants.

To estimate the number of rare benign stopgains in individuals, we collected variants from Phase 3 of the 1000 Genomes Project [2]. We isolated single base-pair stopgain substitutions using ANNOVAR [3] (version 2019Oct24, see URLs). We provided ANNOVAR the coding gene isoforms from the GENCODE Version 33 (Ensembl 99) Basic Gene Annotation Set for the hg38 assembly of the human genome. The set contains 57,727 isoforms, corresponding to 19,644 unique protein-coding genes. The following precedence list was input to ANNOVAR in order to break ties when a variant could be assigned multiple potential annotations:

splicing → exonic → ncRNA → UTR5 → UTR3 → intronic → upstream → downstream → intergenic

Dataset curation

We built two datasets of pathogenic and benign single base-pair stopgain substitutions (Fig. S1). The first dataset, $\mathcal{D}_{\text{original}}$, contains pathogenic and benign variants from HGMD Professional 2019.1 and gnomAD exomes 2.1.1, respectively. The second dataset, $\mathcal{D}_{\text{validation}}$, contains pathogenic variants from HGMD Professional 2020.1 and ClinVar [4] (data downloaded on 07/28/2020, see URLs) and benign variants from gnomAD genomes 3.0. We removed all variants from $\mathcal{D}_{\text{validation}}$ that were already present in $\mathcal{D}_{\text{original}}$.

Variant filtration: Only those variants that were annotated by ANNOVAR as stopgains and that had an allele frequency of $< 1\%$ within both gnomAD exomes 2.1.1 and gnomAD genomes 3.0 were included in the final datasets. Moreover, we only retained HGMD variants tagged as DM and ClinVar variants tagged as Pathogenic. Lastly, any gnomAD variant that was homozygous in at least one individual and that was also listed in HGMD or ClinVar was removed. Heterozygous gnomAD variants that overlapped pathogenic variants were not discarded.

Train-test split for $\mathcal{D}_{\text{original}}$: All variants used by ALoFT and MutPred-LoF were placed in the training set. (ALoFT sources its pathogenic set from HGMD 2016 and benign set of homozygous stopgains from Phase 1 of the 1000 Genomes Project. MutPred-LoF also compiles its pathogenic set from HGMD 2016, but its benign set comes from ExAC 0.3.) The remaining variants were randomly divided between the training and test set to generate a 90%-10% split. Note that $\mathcal{D}_{\text{validation}}$ was used for testing in its entirety.

Features

Zygosity: We labeled variants as either homozygous or heterozygous. Following the protocol described in S-CAP [5], benign variants from gnomAD were labeled as homozygous if and only if they were homozygous in at least one gnomAD individual or if they were located on a sex chromosome, excluding the pseudoautosomal region.

Gene/exon essentiality: (1) We derived a stopgain-specific version of the *oe* (observed/expected) ratio proposed in [6]. Our statistic is computed by dividing the number of observed benign stopgain alleles in the training set by gnomAD's expected number of loss-of-function alleles (see URLs). Each gene and transcript was then assigned a relative *oe* percentile. (2) We obtained RVIS scores per gene from the Genic Intolerance Website (see URLs) and included the data in the `RVIS[pop_maf_0.05%(any)]` column as a feature. Genes not present in the file were given a default RVIS percentile of 50. (3) Two non-exclusive binary features were included for overlapping a dominant and/or recessive OMIM disease gene (see URLs, data downloaded on 06/21/2020). (4) We characterized transcripts and exons as monoclass pathogenic if at least one pathogenic variant, but no benign variants, in the training set overlapped the transcript or exon. For training variants, we masked the effect of the variant itself on these two features. (5) We checked if there was at least one gene isoform that did not contain the variant.

Stopgain-specific features: (1) *Variant location*: We included two absolute distances of the variant from the CDS start and end and a relative location equal to the distance from the CDS start divided by the total CDS length. Similar statistics were also computed for location within the exon. We also included the number of exons in the transcript and the index of the affected exon. Lastly, we specified if the variant was located on an autosomal, X, or Y chromosome. (2) *NMD*: We provided the model with the number of coding base pairs between the last exon-exon junction and the variant (negative values indicate that the variant is downstream of the last exon-exon junction). We define the location of the last exon-exon junction as the most downstream base pair within the second to last exon, and, for transcripts with just one exon, we consider it to be the CDS start. We also quantified the percentage of isoforms in which the variant is more than 50 base pairs upstream of the last exon-exon junction. (3) *Stop codon read-through*: We passed the model a one-hot encoding (with three dimensions) of the premature stop codon created by the mutation. (4) *Alternative translation reinitiation*: We included the distance between the premature stop codon and the next potential start codon (ATG) in any same-strand frame within the sequence. If no start codon was found, this value was set equal to the distance to the CDS end. (5) *Sequence conservation*: phyloP and phastCons scores from the UCSC hg38 100-way Multiz Conservation track were extracted using the pyBigWig Python library. For each variant, we included six scores: the mean score of the upstream region, downstream region, and overlapped exon for both phyloP and phastCons. The upstream region is the coding sequence between the CDS start and the variant, and the downstream region is the coding sequence between the variant and the CDS end. Bases without conservation scores were assigned a default value of 0.

Model training

To train X-CAP, we used the Gradient Boosted model provided by the LightGBM library v3.1.1 [7]. Appropriate hyperparameters and features were identified using fivefold cross-validation on the training set of $\mathcal{D}_{\text{original}}$. Specifically, a grid search over the following hyperparameters was performed:

- `num_iterations`: 100, 500, 1000
- `min_data_in_leaf`: 1, 5, 10, 20
- `max_depth`: 3, 6, 9

We ultimately selected the following hyperparameters: `num_iterations` = 500, `min_data_in_leaf` = 20, `max_depth` = 6. As opposed to previous works we are aware of (including our own) that likely maximized the overall AUROC, these hyperparameters maximized the area under the curve within the clinically relevant high-sensitivity region (hsr-AUROC), averaged across all folds. We then used these hyperparameters to train the model on the full training set.

Model comparison

We compared our method to ALoFT, MutPred-LoF, CADD, DANN, and Eigen. We downloaded the ALoFT classifier from the ALoFT website (downloaded on 04/09/20, see URLs) and ran the tool after lifting over variants to the hg19 assembly using `LiftoverVcf` from the Picard tool suite (see URLs). For each variant, ALoFT provides three probabilities corresponding to whether the variant is benign, dominant pathogenic, or recessive pathogenic. As ALoFT does not explicitly describe a method to assign a pathogenicity score to a variant of particular zygosity, such as all patient variants, we used the following heuristic. Heterozygous variants were assigned a score equal to the dominant pathogenic probability. Homozygous variants were given a score equal to the recessive pathogenic probability plus the dominant pathogenic probability. As part of its pipeline, ALoFT discards stopgain variants that it does not think cause loss of function. Those discarded variants were assigned a score of 0. ALoFT scores across different overlapping transcripts of the same gene were averaged.

We downloaded MutPred-LoF from the MutPred-LoF website (downloaded on 04/21/2020, see URLs). MutPred-LoF accepts the output of ANNOVAR's `coding_change.pl` script as input, so we ran that script with the ANNOVAR version and gene set listed above. Because of the long running time of MutPred-LoF (MutPred-LoF is 84 times slower than X-CAP on 1000 variants; Table S1), we randomly sampled 1000 variants when evaluating this classifier on $\mathcal{D}_{\text{original}}$ and $\mathcal{D}_{\text{validation}}$.

CADD, DANN, and Eigen scores were taken from dbNSFP v4.1a [8]. The `annotate_variation.pl` script provided in the ANNOVAR suite was used to download dbNSFP annotations, and the `table_annovar.pl` script was used to look up annotations for specific variants. Variants without provided scores were assigned a default score of 0.

Evaluation on exomes

We compiled putatively benign stopgains from a control population ($N = 480$) in an Inflammatory Bowel Disease Exome Sequencing Study (dbGaP Study Accession: phs001076.v1.p1, consent group: GRU) [9]. All classifiers were tested on rare variants that none had previously seen.

We also collected variant call files of patients in the Deciphering Developmental Disorders (DDD) project [10] who harbored one stopgain and no other rare mutations in the causal gene. We isolated exomes in which the causal stopgain was not seen by any of the compared classifiers.

URLs

- ANNOVAR: `https://annovar.openbioinformatics.org/en/latest/`
- ClinVar: `https://ftp.ncbi.nlm.nih.gov/pub/clinvar/vcf_GRCh38/`
- gnomAD *oe*
  - per transcript values: `https://storage.googleapis.com/gcp-public-data--gnomad/release/2.1.1/constraint/gnomad.v2.1.1.lof_metrics.by_transcript.txt.bgz`
  - per gene values: `https://storage.googleapis.com/gcp-public-data--gnomad/release/2.1.1/constraint/gnomad.v2.1.1.lof_metrics.by_gene.txt.bgz`
- RVIS: `https://genic-intolerance.org/data/RVIS_Unpublished_ExACv2_March2017.txt`
- OMIM gene map data: `https://www.omim.org/search/advanced/geneMap`
- ALoFT: `http://aloft.gersteinlab.org/`
- Picard: `https://broadinstitute.github.io/picard/`
- MutPred-LoF: `http://mutpred2.mutdb.org/mutpredlof/`
- shap: `https://github.com/slundberg/shap`

References

1. Stenson, P. D. *et al.* The Human Gene Mutation Database (HGMD®): optimizing its use in a clinical diagnostic or research setting. *Human Genetics* **139,** 1197–1207 (2020).
2. The 1000 Genomes Project Consortium. A global reference for human genetic variation. *Nature* **526,** 68–74 (2015).
3. Wang, K., Li, M. & Hakonarson, H. ANNOVAR: functional annotation of genetic variants from high-throughput sequencing data. *Nucleic Acids Research* **38,** e164 (2010).
4. Landrum, M. J. *et al.* ClinVar: improving access to variant interpretations and supporting evidence. *Nucleic Acids Research* **46,** D1062–D1067 (2018).
5. Jagadeesh, K. A. *et al.* S-CAP extends pathogenicity prediction to genetic variants that affect RNA splicing. *Nature Genetics* **51,** 755–763 (2019).
6. Karczewski, K. J. *et al.* The mutational constraint spectrum quantified from variation in 141,456 humans. *Nature* **581,** 434–443 (2020).
7. Ke, G. *et al. LightGBM: A highly efficient gradient boosting decision tree* in *Advances in Neural Information Processing Systems* (2017), 3146–3154.
8. Liu, X., Li, C., Mou, C., Dong, Y. & Tu, Y. dbNSFP v4: a comprehensive database of transcript-specific functional predictions and annotations for human nonsynonymous and splice-site SNVs. *Genome Medicine* **12,** 1–8 (2020).
9. Beaudoin, M. *et al.* Deep resequencing of GWAS loci identifies rare variants in CARD9, IL23R and RNF186 that are associated with ulcerative colitis. *PLoS Genetics* **9,** e1003723 (2013).
10. Firth, H. V. & Wright, C. F. The Deciphering Developmental Disorders (DDD) study. *Developmental Medicine and Child Neurology* **53,** 702–703 (2011).

11. Lundberg, S. M. *et al.* From local explanations to global understanding with explainable AI for trees. *Nature Machine Intelligence* **2,** 56–67 (2020).

2 Supplementary Figures

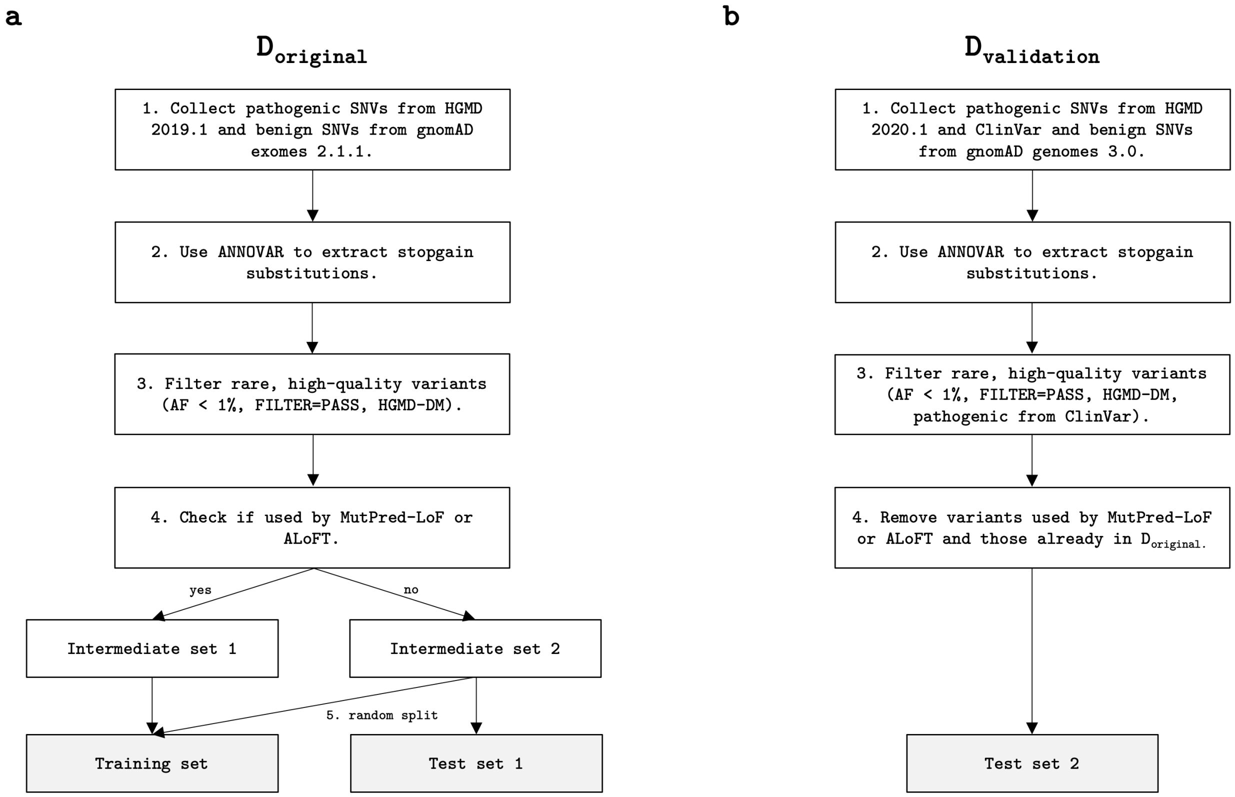

	variants	genes
pathogenic	22,584	2,514
benign	144,220	17,662

	variants	genes
pathogenic	2,510	1,038
benign	16,027	9,035

	variants	genes
pathogenic	10,295	1,921
benign	53,622	15,423

Figure S1: Pipeline for curating datasets. Schematic of the process used to generate two datasets: **(a)** $\mathcal{D}_{\text{original}}$, which contains both training and test splits, and **(b)** $\mathcal{D}_{\text{validation}}$, which is only used for testing. Crucially, we only use rare variants that have an allele frequency (AF) $< 1\%$ to train and evaluate the model. Under each shaded dataset, we tabulate the number of variants in the two pathogenicity classes and the number of genes they appear in. Note that a gene may be counted in both rows. SNV, single-nucleotide variant.

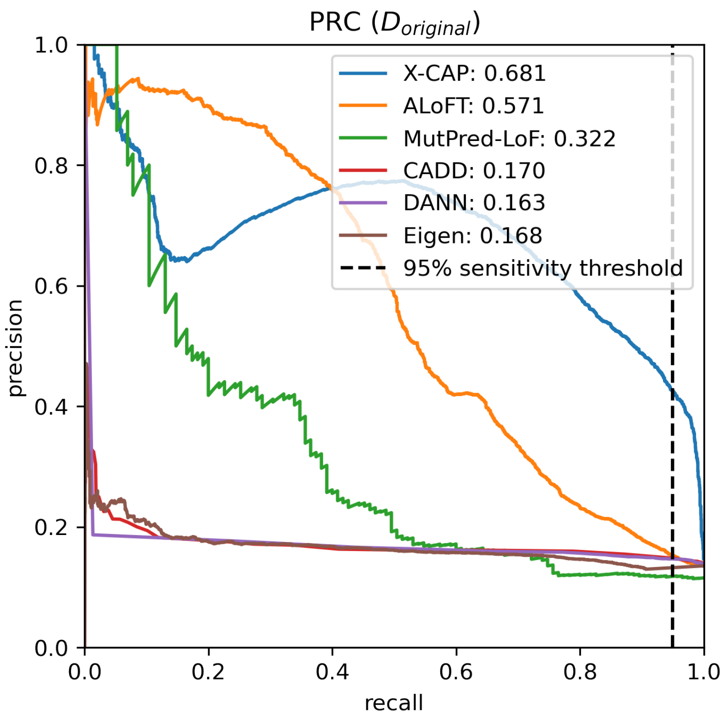

Figure S2: Precision-recall curve for $\mathcal{D}_{\mathbf{original}}$. Analogously to Fig. 3, we plot the precision-recall curves (PRC) and display the associated AUPRC values for each model on the test set of $\mathcal{D}_{\text{original}}$. X-CAP appreciably increases the previous best AUPRC by 19%. In particular, at 95% recall (which is equivalent to sensitivity), X-CAP has more than twice the precision of any other classifier.

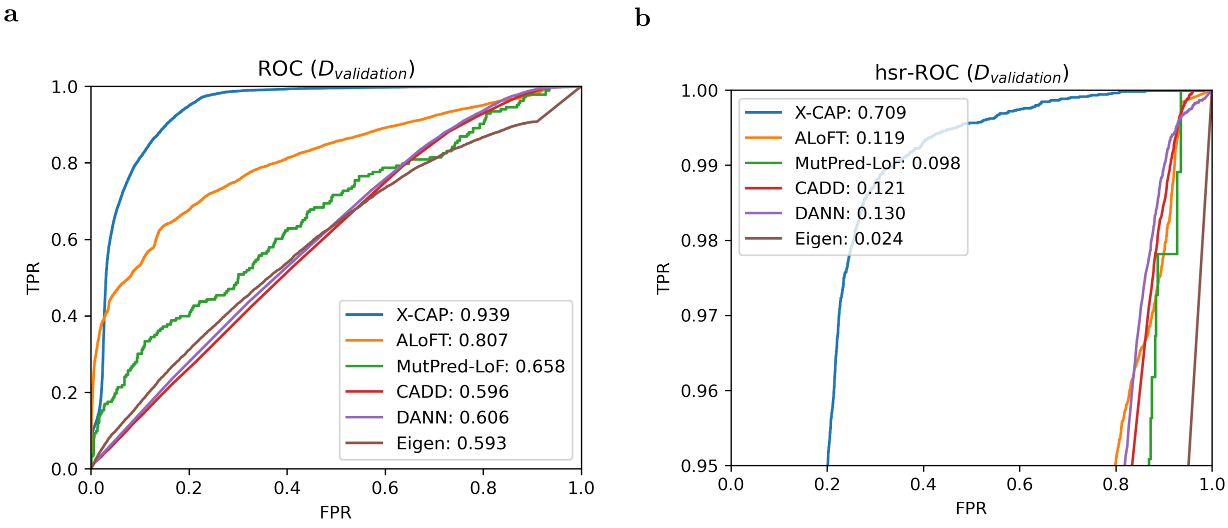

Figure S3: X-CAP generalizes well. We compare each model's performance on an independently generated dataset, $\mathcal{D}_{\text{validation}}$. Similar to Fig. 3, we plot both the **(a)** full and **(b)** high-sensitivity region ROC curves. X-CAP increases the AUROC by 16% and the hsr-AUROC by 490%. At 95% sensitivity, X-CAP correctly classifies 79.9% of benign stopgain variants, whereas ALoFT, the next best model, does so for only 20.1%. This represents a 4-fold improvement.

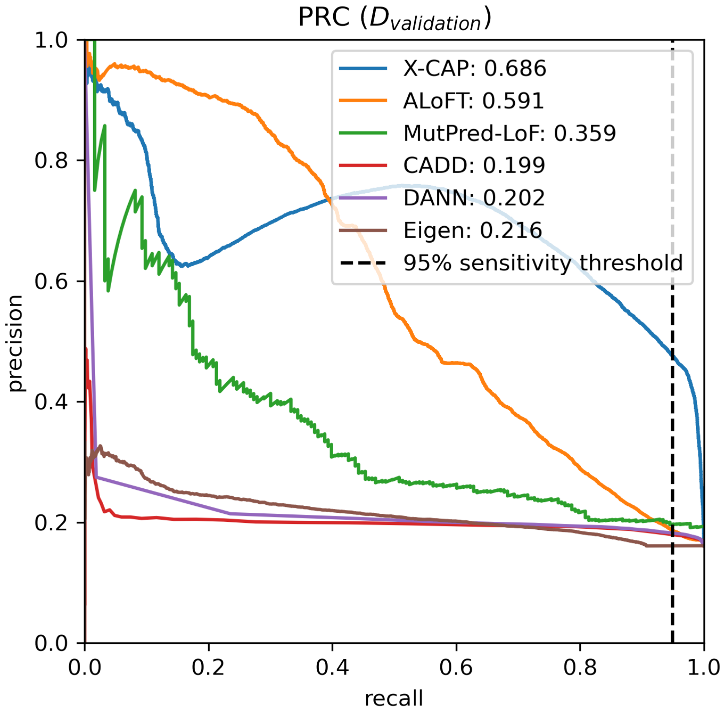

Figure S4: Precision-recall curve for $\mathcal{D}_{\textbf{validation}}$. The precision-recall curves and associated AUPRC values for each model on $\mathcal{D}_{\text{validation}}$ are plotted. The models' rankings on the AUPRC metric closely mirror their rankings on the AUROC metric (compare this plot to Fig. S3a). X-CAP again boasts the highest AUPRC.

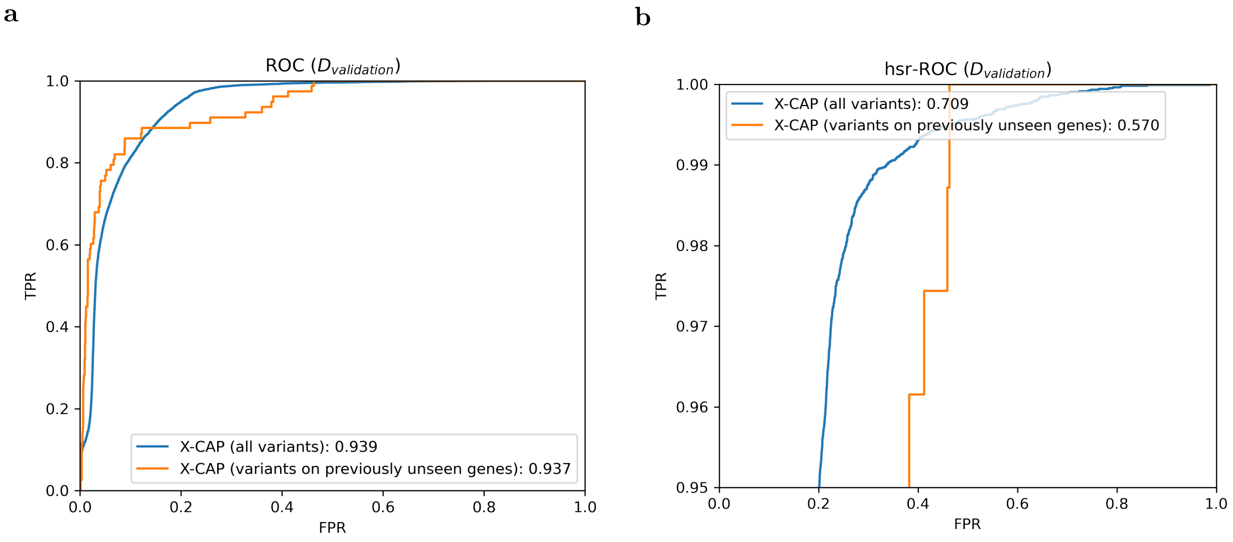

Figure S5: X-CAP's performance on unseen genes. We compare X-CAP's performance on the full set of variants in $\mathcal{D}_{\text{validation}}$ versus only those variants located in previously unseen genes (i.e. genes which contained no pathogenic or benign variants in the training set). We plot both the **(a)** full and **(b)** high-sensitivity region ROC curves. While variants in unseen genes present a challenge at high sensitivity, our performance on them is still much better than the total performance of all other tools (compare to Fig. S3). The subset of variants on previously unseen genes consists of 78 pathogenic and 1887 benign variants across 32 pathogenic and 783 benign genes, respectively.

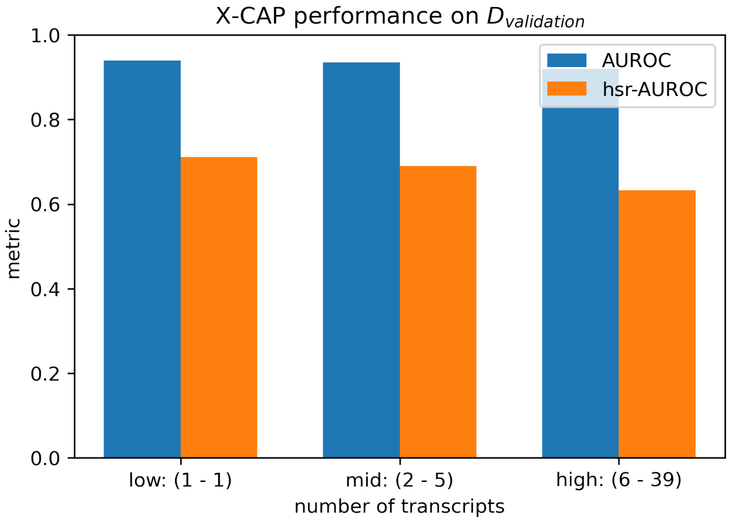

Figure S6: X-CAP's performance is robust to the number of transcripts that a variant overlaps. We segmented the variants in $\mathcal{D}_{\text{validation}}$ into three approximately equally sized bins, according to the number of transcripts that each variant overlaps. The first bin contains variants overlapping one transcript, the second contains variants overlapping two to five transcripts, and the third contains variants overlapping at least six transcripts. Across all three bins, the AUROC and hsr-AUROC metrics are quite similar.

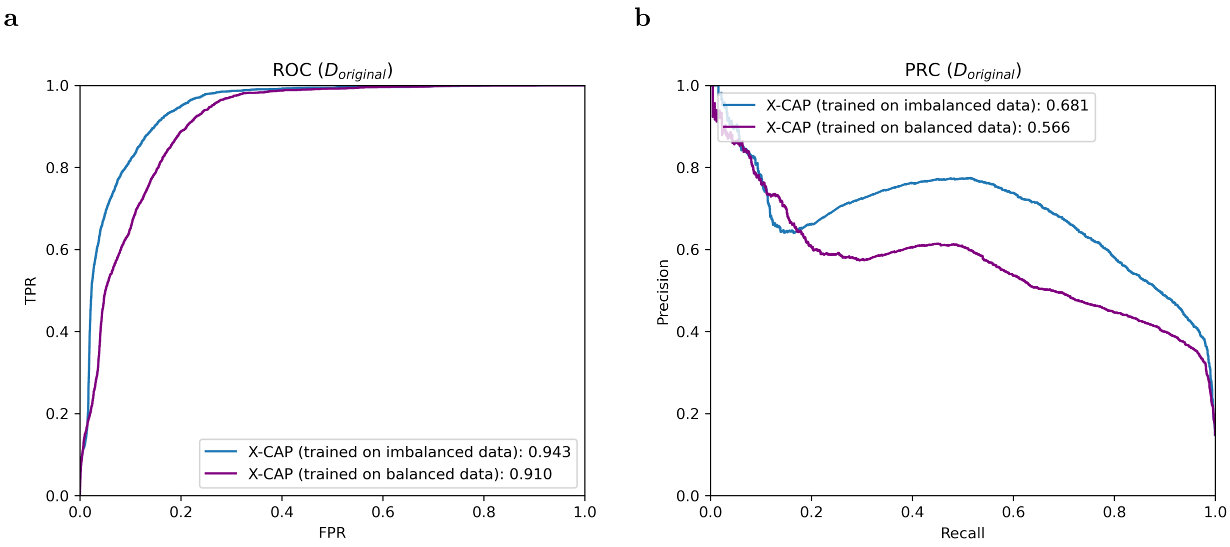

Figure S7: X-CAP is robust enough to benefit from a larger, albeit imbalanced, dataset. X-CAP is trained on an imbalanced dataset (144,220 benign v. 22,584 pathogenic stopgains). If we balance our training data by randomly subsampling our benign set to be of the same size as our pathogenic set, performance decreases. In **(a)**, we plot the ROC curves and associated AUROC values when evaluated on the test of $\mathcal{D}_{\text{original}}$, and in **(b)**, we plot the precision-recall curves and associated AUPRC values. The model performs slightly better when trained on the larger, imbalanced training set.

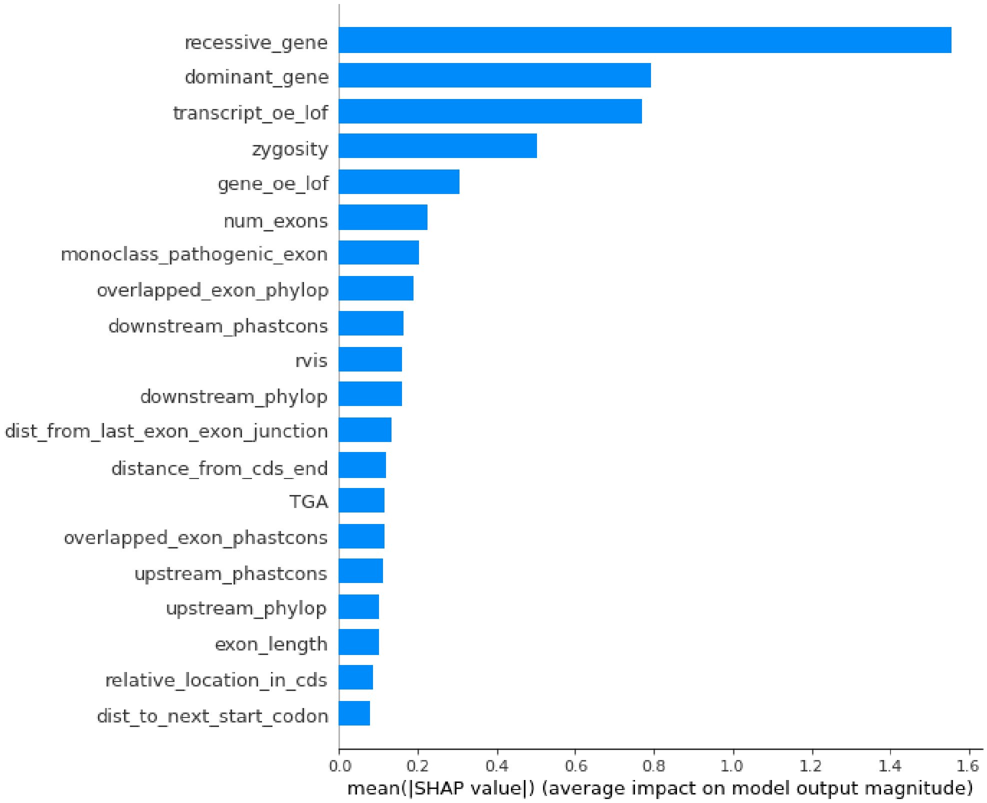

Figure S8: X-CAP feature importance values. The top 20 features, according to their Shapley feature importance values on the training set of $\mathcal{D}_{\text{original}}$, are listed in order. Briefly, Shapley values measure the average contribution each feature makes to the model's predictions across all possible permutations of other features [11]. Many novel features, notably zygosity, which are introduced in X-CAP (compare to Table 1) have high Shapley feature importance values. This plot was generated using the shap Python package (see URLs).

a **b**

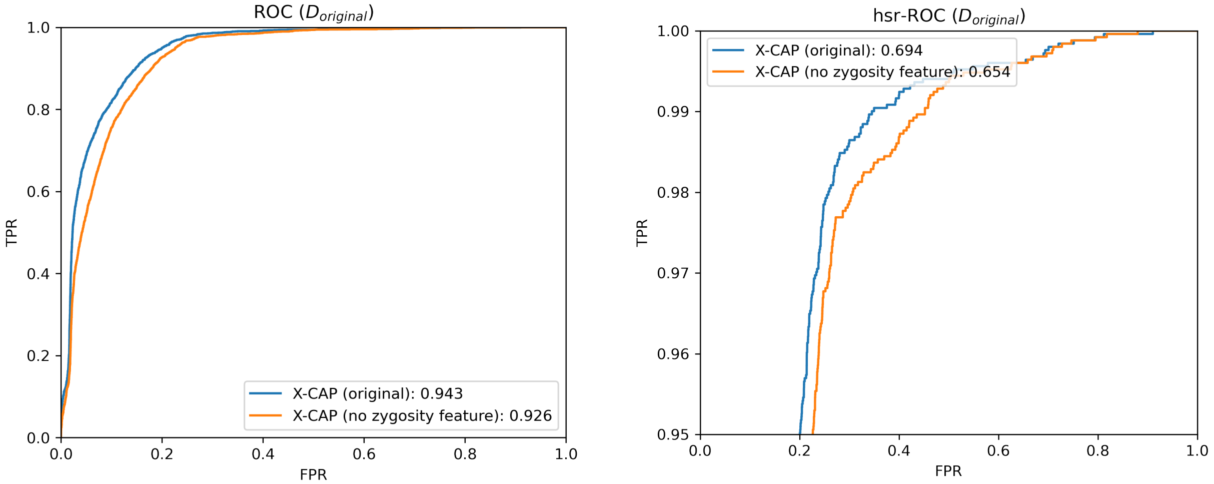

Figure S9: Including our zygosity heuristic as a feature improves performance. We compare the performance of X-CAP when zygosity is included as a feature, as opposed to when it is not, on the test set of $\mathcal{D}_{\text{original}}$. We plot **(a)** the full ROC curves and **(b)** the high-sensitivity region ROC curves.

3 Supplementary Tables

Table S1: X-CAP runs faster than competitors

model	mean (hh:mm:ss)	standard deviation (hh:mm:ss)
X-CAP	00:03:20	00:00:27
ALoFT	00:10:21	00:01:23
MutPred-LoF	04:42:22	00:27:28

Running time of each classifier on a random sample of 1000 stopgain variants, averaged over five trials. All classifiers were run on a machine with 32 GB of RAM and 8 Intel Xeon E-5 1620 v3 cores, each with 3.5 GHz clock speed. CADD, DANN, and Eigen were excluded from this comparison because their scores have been pre-computed and stored in a lookup table for easy access. The abbreviation hh:mm:ss stands for hours:minutes:seconds.
